# Prediction of fetal acidemia in placental abruption

**DOI:** 10.1186/1471-2393-13-156

**Published:** 2013-08-01

**Authors:** Yoshio Matsuda, Masaki Ogawa, Jun Konno, Minoru Mitani, Hideo Matsui

**Affiliations:** 1 Department of Obstetrics and Gynecology, Tokyo Women’s Medical University, Kawada-cho, 8-1, Shinjuku-ku, Tokyo 162-8666, Japan; 2 Perinatal Medical Center, Tokyo Women’s Medical University, Kawada-cho, 8-1, Shinjuku-ku, Tokyo 162-8666, Japan; 3 Department of Obstetrics and Gynecology, International University of Health and Welfare Hospital, 537-3, Iguchi, Nasushiobara-City, Tochigi-Prefecture 329-2763, Japan

**Keywords:** Bradycardia, Fetal acidemia, Fetal heart rate monitoring, Placental abruption, Severe abruption score

## Abstract

**Background:**

To determine the major predictive factors for fetal acidemia in placental abruption.

**Methods:**

A retrospective review of pregnancies with placental abruption was performed using a logistic regression model. Fetal acidemia was defined as a pH of less than 7.0 in umbilical artery. The severe abruption score, which was derived from a linear discriminant function, was calculated to determine the probability of fetal acidemia.

**Results:**

Fetal acidemia was seen in 43 survivors (43/222, 19%). A logistic regression model showed bradycardia (OR (odds ratio) 50.34, 95% CI 11.07 – 228.93), and late decelerations (OR 15.13, 3.05 – 74.97), but not abnormal ultrasonographic findings were to be associated with the occurrence of fetal acidemia. The severe abruption score was calculated for the occurrence of fetal acidemia, using 6 items including vaginal bleeding, gestational age, abdominal pain, abnormal ultrasonographic finding, late decelerations, and bradycardia.

**Conclusions:**

An abnormal FHR pattern, especially bradycardia is the most significant risk factor in placental abruption predicting fetal acidemia, regardless of the presence of abnormal ultrasonographic findings or gestational age.

## Background

Placental abruption is one of the serious complications of late pregnancy, because it leads to both poor maternal and fetal/neonatal outcome [1]. The diagnosis of placental abruption usually depends on the clinical manifestations, and confirmed the placental detachment after delivery [2,3]. The usefulnesses of ultrasonography [4,5] and fetal heart rate (FHR) monitoring have been reported as the adjunctive diagnosis, and they are both widely used for this purpose. But controversy exists as to whether ultrasonographic findings are useful in determining the diagnosis, management and the relation to fetal outcome. On the other hand, abnormal FHR patterns are also common in severe cases [6], reflecting uteroplacental insufficiency resulting from interruption of placental blood flow [7]. However; few studies have compared the simultaneous use of these two modalities in cases of placental abruption.

Pathological acidemia is defined as a pH of less than 7.0 and it is an objective measure of intrapartum hypoxia-ischemia and has been correlated with the occurrence of hypoxic ischemic encephalopathy (HIE) [8]. It has been reported that placental abruption is as a reliable factor for fetal acidemia as well as FHR abnormality in both preterm and term infants [9,10], although the rate of the occurrence of fetal acidemia has not been well described in placental abruption.

The purpose of this study was therefore to determine the important factors and to establish the predictive score for acidemia in placental abruption, by evaluating clinical assessments as well as adjunctive laboratory tests such as ultrasonographic findings and FHR patterns.

## Methods

The approval of the institutional review board (Tokyo Women’s Medical University) was obtained before the start of this retrospective, case–control study. All singleton births, born between 24 and 40 weeks of gestation between January, 1st, 2009, and December 31st, 2009 were included. The medical records of mothers and neonates in the 94 institutes where these infants were born, comprising the Perinatal Research Network in Japan (PRNJ) listed in the ‘Acknowledgements’ were reviewed.

Gestational age was determined based on the mother’s last menstrual period and first and second trimester obstetric ultrasonography. More than 30 variables were assessed, including pregestational and perinatal factors. Preeclampsia/superimposed preeclampsia was diagnosed according to the criteria of National High Blood Pressure Education Program Working Group on High Blood Pressure in Pregnancy [11]. Details of the diagnosis, onset place, time from onset of symptoms to admission/delivery, and clinical management of any relevant condition were recorded.

The diagnosis of placental abruption was based primarily on the one or more of the following clinical manifestations: vaginal bleeding, abdominal pain, uterine tenderness, with/without an abnormal FHR tracing [2], and confirmed the placental detachment after delivery. The presence of hematoma during a cesarean section or coagulation/massive genital bleeding during vaginal delivery was considered to indicate placental detachment. Women found to have a healthy placenta, and those with chronic abruption, were excluded from this study. Chronic abruption-oligohydramnios sequence (CAOS) was defined by the following criteria: (1) clinically significant vaginal bleeding in the absence of placenta previa or other identifiable source of bleeding, (2) amniotic fluid volume initially documented as normal and (3) oligohydramnios (amniotic fluid index < or = 5) eventually developing without concurrent evidence of ruptured membranes [12].

Ultrasonographic findings were reported as follows; preplacental collection under the chorionic plate, increased heterogenous placental thickness (more than 5 cm), retroplacental collection, marginal hematoma, subchorionic hematoma, or intra-amniotic hematoma [5]. FHR patterns were defined as abnormal when one of the following patterns was detected: persistent late decelerations, severe atypical deceleration, prolonged deceleration, or bradycardia [13]. The same patterns were also used for the assessment of preterm fetuses. A baseline FHR of less than 100 bpm was noted as bradycardia (>3 mins) [13]. This includes FHR detected only by hand-held Doppler fetal heart detectors or auscultation in some cases.

The live fetuses were delivered by cesarean section because of abnormal FHR patterns or maternal indications, such as massive genital bleeding. At delivery, the umbilical cord was double clamped. 0.2 ml of arterial blood was collected on ice for blood gas analysis. An abnormal umbilical arterial pH (fetal acidemia) was defined as a value of less than 7.0 [14].

An adverse outcome for the neonate was defined as the occurrence of death before hospital discharge or a diagnosis of cerebral palsy. The outcome of pregnancy was classified as poor (cases) and good (controls), with a poor outcome defined as a fetal arterial cord blood pH of less than 7.0, irrespective of neonatal outcome. The results were expressed as mean ± standard deviation (SD), medians with range, or numbers with the percentage. Statistical analyses were performed with the Statflez 6.0 software package (Archtech Co., Ltd., Osaka, Japan. URL:http://www.statflex.Net/) and were carried out using the chi-square test, Fisher’s exact probability test, and the Mann–Whitney U test. *p* values of less than 0.05 were considered to be significant. The odds ratio (OR) and 95% confidence intervals (CI) were calculated to estimate the relative risk between cases and controls with regard to risk factors for fetal acidemia. They were compared in both univariate and multivariate analyses. Logistic regression models were used to assess confounding effects and to construct a discriminant function. Receiver operating characteristic (ROC) curves were constructed to evaluate the relationship between the sensitivity and false positive rate (1-specificity) for the indicators of fetal acidemia. The optimal cut-off point was determined to be the point corresponding to the highest sensitivity in relation to the highest specificity.

## Results

There were 355 patients complicated by placental abruption. The overall number of deliveries in 94 institutes was 54,628 and the rate of placental abruption was 0.65%. Eighty-nine fetuses were dead on admission (intrauterine fetal death), while the remaining 266 fetuses were alive. The latter cases were included in the analysis for this study. Although eighty three cases were not recorded mainly due to the emergency nature of the situation without CTG recordings, rapid frequent contractions with no resting tone were observed in 72 out of the remaining 183 cases. The mean gestational age at delivery was 34.1 ± 3.4 weeks (preterm: 200 patients, term: 66 patients). In spite of resuscitation, an Apgar score of 0 at five minutes was seen in 7 cases, which resulted in adverse outcome.

Abnormal ultrasonographic findings were observed in 183 patients (68.8%), mainly indicating retroplacental anechoicity (121 cases), and increased placental thickness (117 cases).

Abnormal FHR patterns were observed in 166 patients (62.4%). The main abnormal FHR results were as follows: persistent late decelerations, 51 cases and prolonged deceleration, 86 (including by auscultation) cases. Cesarean section was performed in 256 patients (96.2%). Of these patients, a neonatal adverse outcome was seen in 18 cases. On the other hand, in the remaining 10 patients who delivered vaginally, a neonatal adverse outcome was not observed.

As the data regarding the umbilical artery pH in 44 cases were not obtained, we used the data from the remaining 222 cases, and fetal acidemia was seen in 43 (19%). These patients were divided into two groups: poor outcome group (case, *n* = 43), and good outcome group (control, *n* = 179). Neonatal adverse outcome cases included 22 patients and there was a significant difference between cases and controls in the occurrence of neonatal adverse outcome (14/ 43, 32.6% vs. 8/179, 4.5%, p < 0.001).

The clinical demographics of the cases are given in Table 1. Risk factors for cases and controls based on the occurrence of perinatal events in placental abruption were analyzed. In the univariate analysis, abnormal FHR patterns, bradycardia, and maternal unstable condition necessitating the blood transfusion were more frequent in cases.

**Table 1:** Results of univariate analysis in terms of risk factors of fetal acidemia

	**Case (N = 43)**	**Control (N = 179)**	***p***
**Primipara**	**24 (55.8%)**	**75 (41.9%)**	**0.14**
**Maternal transfer**	**28 (65.1%)**	**109 (60.9)**	**0.742**
**Preeclampsia/eclampsia or chronic hypertension**	**16 (37.2%)**	**42 (23.5%)**	**0.104**
**Vaginal bleeding**	**21 (48.8%)**	**98 (54.8%)**	**0.596**
**Abdominal pain**	**20 (46.5%)**	**57 (31.8%)**	**0.104**
**Abnormal ultrasonographic finding**	**35 (81.4%)**	**128 (71.5%)**	**0.257**
**Abnormal FHR patterns**	**42 (97.7%)**	**98 (54.7%)**	**<0.0001**
**Bradycardia**	**32 (74.4%)**	**42 (23.5%)**	**<0.0001**
**Persistent late decelerations**	**9(20.9%)**	**34 (19.0%)**	**0.919**
**Cesarean section**	**41 (95.3%)**	**171 (95.5%)**	**0.993**
**Gestational age at delivery (weeks)**	34(24–39)*	35 (24–41)*	**0.07**
**Gestational age at delivery <35 wks**	**19 (44.2%)**	**99 (55.3%)**	**0.253**
**Birth weight (g)**	1997 (517–3504)*	2062 (774–3450)*	**0.107**
**Male**	**22 (51.2%)**	**108 (60.3%)**	**0.568**
**Apgar score (1 min)**	2(0–6)*	6 (0–9)*	**<0.0001**
**Apgar score (5 min)**	5 (0–9)*	8 (0–10)*	**<0.0001**
**Blood transfusion**	**21 (48.8%)**	**37 (20.7%)**	**0.006**

*represents median (min-max).

Factors affecting umbilical arterial pH were determined by multiple regression analysis (Table 2). As a result, four factors significantly affected umbilical arterial pH; partial regression coefficients of bradycardia, persistent late decelerations, abnormal ultrasonographic findings, and abdominal pain were −0.256, -0.133, -0.054, and −0.047, respectively.

**Table 2:** Results of multiple regression analysis affecting umbilical arterial pH

**Potential predictors**	**Partial regression coefficient**	**95% CI**	***p***
**Bradycardia**	**−0.256**	**−0.302 – -0.210**	**<0.0001**
**Persistent late decelerations**	**−0.133**	**−0.186 - -0.08**	**<0.0001**
**Gestational week at delivery**	**0.006**	**−0.0002 – 0.011**	**0.059**
**Abnormal ultrasonographic findings**	**−0.054**	**−0.099 – -0.009**	**0.019**
**Abdominal pain**	**−0.047**	**−0.089 – -0.006**	**0.026**
**Vaginal bleeding**	**−0.021**	**−0.061 – 0.02**	**0.318**

Using a logistic regression model, bradycardia (OR (odds ratio) 50.34, 95%CI 11.07 – 228.93), and persistent late decelerations (OR 15.13, 3.05 – 74.97), but not abnormal ultrasonographic findings were to be associated with the occurrence of fetal acidemia (Table 3).

**Table 3:** Results of multivariate analysis in terms of risk factors for fetal acidemia

**Potential predictors**	**Odds ratio**	**95% CI**	***p***
**Bradycardia**	**50.34**	**11.07 – 228.93**	**<0.0001**
**Persistent late decelerations**	**15.13**	**3.05 – 74.97**	**<0.0001**
**Gestational week at delivery < 35 weeks**	**1.82**	**0.81 – 4.17**	**0.152**
**Abnormal ultrasonographic findings**	**2.01**	**0.75 – 5.39**	**0.164**
**Abdominal pain**	**1.70**	**0.73 – 3.92**	**0.216**
**Vaginal bleeding**	**1.47**	**0.64 – 3.44**	**0.362**

**Figure 1:**
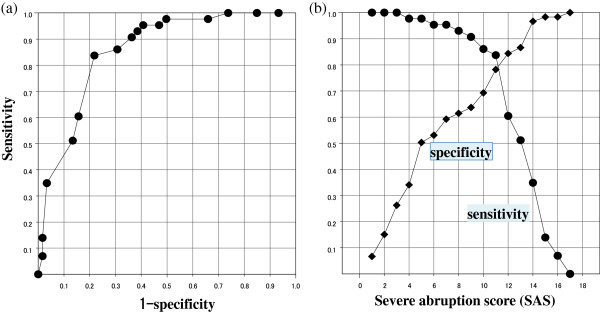
**Severe abruption score (SAS) in cases with placental abruption. (a)** Receiver operating characteristics curve. **(b)**. Sensivity and specificity curve.

**Figure 2:**
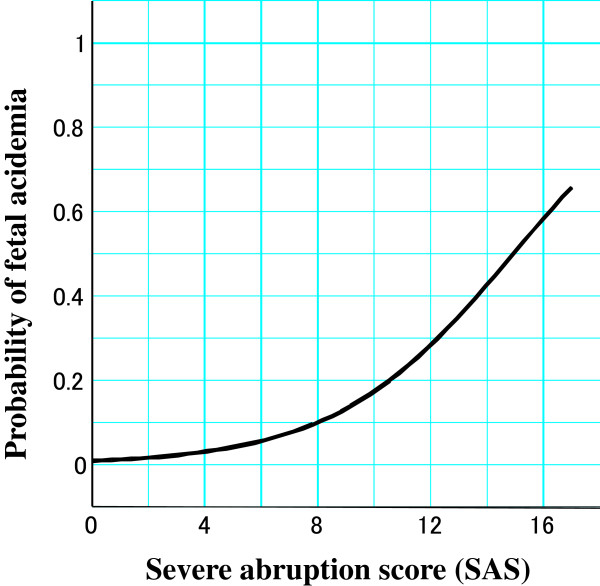
Relationship between the severe abruption score (SAS) and the probability of fetal acidemia in the cases of placental abruption.

The statistically significant factors identified by the multiple logistic regression analysis were subjected to stepwise regression analysis to construct a linear discriminant function: A + 2B + 2C + 2D + 7E + 10 F, where A was vaginal bleeding (0, no; 1, yes), B was gestational age less than 35 weeks (0, no; 1, yes), C was abdominal pain (0, no; 1, yes), D was abnormal ultrasonographic finding (0, no; 1, yes), E was persistent late decelerations (0, no; 1, yes), and F was bradycardia (0, no; 1, yes). This discriminant function was named as ‘severe abruption score’ (SAS). A ROC curve is shown in Figure 1a. The area under the curve (AUC) was 0.855. The sensitivity and specificity curve showed that the optimal cut-off value for the SAS was 11 [sensitivity: 83.7%; specificity: 78.2%; positive predictive value (PPV): 43.6%; negative predictive value (NPV): 93.8%] to 12 [sensitivity: 69.8%; specificity: 81%; positive predictive value (PPV): 48%; negative predictive value (NPV): 95.2%], as shown in Figure 1b. Then, logistic regression analysis was performed to make clear the relationship between the SAS and the probability of fetal acidemia in Figure 2. When this score was 15, the probability of fetal acidemia was 0.5.

## Discussion

We have evaluated clinical symptoms, ultrasonographic findings and FHR patterns in placental abruption from the analysis of relatively large number of cases and showed that ‘severe abruption score’ is useful for prediction for fetal acidemia in placental abruption, although this score is derived from the retrospective diagnosis of abruption and cannot be used before delivery.

Because the etiology of placental abruption is heterogeneous and speculative, and clinical abruption is the final culmination of a long-standing disease process within the placenta, [15] the diagnosis of placental abruption in this study was based on the clinical manifestations, and the placental detachment was confirmed after delivery. Therefore, the clinical background of patients in the present study may be more severe than some other studies [3,16]. As a result, the percentage of maternal and fetal/neonatal morbidity and mortality was high.

The association between placental abruption and the occurrence of infant neurodevelopmental disorders has been confirmed [17]. Abruption increases hypoxic insults through two mechanisms [7]. The separation of the placenta from the maternal blood supply, depending on the extent, decreases the placental surface area and thus oxygen exchange. In addition, there is often a marked increase in the frequency, and occasionally, duration of contractions, which prolongs the interval during which oxygen delivery to the intervillous space is interrupted. Since placental abruption is mechanism of fetal hypoxia/acidosis, placental abruption is one of the most important factors for fetal acidemia [9,10]. In severe cases, the characteristic FHR patterns will be persistent late decelerations, prolonged deceleration, and bradycardia may occur. In the present study, we have defined pathological acidemia as an umbilical arterial pH less than 7.0, which has been linked to the occurrence of HIE [8], and have shown that bradycardia is the major risk factor in placental abruption predicting fetal acidemia, regardless of the presence of abnormal ultrasonographic findings or gestational age.

On the other hand, a severe abruption score derived by discriminant function was calculated for the probability of fetal acidemia as mentioned above. As shown in Figure 1b, optimal cut-off value for severe abruption score was estimated to be 11–12. Even if fetal acidemia was present, there is a possibility for intact survival, if the prompt and aggressive therapy such as brain hypothermia therapy is started as soon as possible [18]. Therefore, this score for confirmed cases might be useful for predicting the need for neonatal intensive therapy soon after delivery.

Moreover, this score represents not only the possibility of prediction for fetal acidemia, but also highlights the significance of each item, thus facilitating clinical diagnosis in placental abruption. The amount of vaginal bleeding correlates poorly with the degree of placental separation and does not serve as a useful marker of impending fetal risk. Even small amounts of vaginal bleeding in the setting of abdominal pain and uterine contractions, we should make close maternal and fetal evaluation for placental abruption [19].

As an adjunctive diagnostic method for placental abruption, the role of ultrasonography is not large. Glantz and colleagues, in a retrospective cohort study, found that the sensitivity, specificity, and positive and negative predictive values of ultrasonography for placental abruption were 24%, 96%, 88% and 53%, respectively and stressed that a negative ultrasonogram did not rule out an abruption [20]. Jaffe and colleagues also found that ultrasonography identified only 50% of abruptions confirmed by pathology [21]. In the present study, abnormal ultrasonographic findings were observed in 183 patients (68.8%). This frequency was slightly high compared to other studies, which might be reflected by the collection of more severe cases. However, using a logistic regression model, abnormal ultrasonographic finding was not to be associated with the occurrence of fetal acidemia. Nyberg and colleagues, in a retrospective review of 69 cases of abruption, found that fetal mortality correlated with the worse prognosis occurring in retroplacental abruption [22]. They did not analyze the relationship between ultrasonographic findings and an adverse outcome of the neonate including neonatal/infantile death before hospital discharge or a diagnosis of cerebral palsy.

There are several limitations in the present study. First, this study has been done by a retrospective fashion; therefore, further study is warranted to prove the usefulness of this score prospectively. Second, as a severe abruption score is based only on cases where a diagnosis of abruption was confirmed according to placental appearance just after delivery and is designed to be used immediately after delivery, this score should be used with caution. Finally, it is important to understand that this score is influenced by the sample size. The prognosis of the babies born following placental abruption will be reported separately.

## Conclusions

In conclusion, an abnormal FHR pattern, especially bradycardia is the most significant risk factor in placental abruption predicting fetal acidemia, and is more valuable than ultrasound in predicting outcome in placental abruption during pregnancy.

### Details of ethics approval

The procedures of the study received ethics approval from the institutional ethics committee responsible for human experimentation (Tokyo Women’s Medical University. Date of approval: 2011/08/22, reference number: 2301).

## Competing interests

The authors report no conflicts of interests.

## Authors’ contributions

Conception: JK, YM, MO, MM, HM. Planning: JK, YM, MO, MM. Carrying out: JK, YM, MO, MM. Analyzing: JK, YM, MO. Writing up: JK, YM, MO, HM. All authors read and approve the final manuscript.

## Pre-publication history

The pre-publication history for this paper can be accessed here:

http://www.biomedcentral.com/1471-2393/13/156/prepub
